# Predictive Factors for the Prognosis of Alcoholic Liver Cirrhosis

**DOI:** 10.3390/medicina58121859

**Published:** 2022-12-16

**Authors:** Anca Trifan, Horia Minea, Adrian Rotaru, Carol Stanciu, Remus Stafie, Ermina Stratina, Sebastian Zenovia, Robert Nastasa, Ana-Maria Singeap, Irina Girleanu, Cristina Muzica, Laura Huiban, Tudor Cuciureanu, Stefan Chiriac, Catalin Sfarti, Camelia Cojocariu

**Affiliations:** 1 Department of Gastroenterology, Grigore T. Popa University of Medicine and Pharmacy, 70015 Iasi, Romania; 2 Institute of Gastroenterology and Hepatology, “St. Spiridon” University Hospital, 700111 Iasi, Romania

**Keywords:** alcoholic liver cirrhosis, mortality, infections, variceal bleeding, hepatorenal syndrome

## Abstract

Alcoholic liver cirrhosis (ALC) is a disease with multiple complications and is associated with poor prognosis and significant mortality. Identifying risk factors associated with a poor outcome is important to ensure effective treatment and increase life expectancy. We aimed to evaluate the predictive values of complications regarding mortality in ALC. We retrospectively analyzed 1429 patients with ALC hospitalized between January 2019 and April 2022 at the Institute of Gastroenterology and Hepatology Iasi. The electronic medical records were interrogated to obtain information about demographic data, complications, comorbidities, and prognostic scores: MELD-Na (model for end-stage liver disease-sodium) and CTP (Child–Turcotte–Pugh). Based on uni- and multivariate analysis, independent predictors of mortality were identified. The mean age at diagnosis was 56.15 ± 11.49 years with a ratio of 2:1 in favor of males. There were 296 deaths (20.8%), most of them during the first hospitalization (208/14.6%). It was observed during the univariate analysis that complications of the disease negatively affected the survival rate, significant values being related to infections (sepsis; OR = 21.98; *p* < 0.001; spontaneous bacterial peritonitis (SBP) (OR = 11.94; *p* < 0.001) and hepatorenal syndrome (HRS) (OR = 9.35; *p* < 0.001). The independent predictors, confirmed by multivariate analysis, were the association of variceal bleeding, infections, and hepatic encephalopathy or ascites, each combination being responsible for two out of 10 of the deaths during the first admission. The prognosis of the disease was negatively influenced by the worsening of liver dysfunction and the appearance of complications. The main predictors of mortality were infections, hepatic encephalopathy, variceal bleeding, and hepatorenal syndrome. Improving compliance and strict application of specific follow-up and treatment strategies could contribute to a better prognosis of patients with alcoholic liver cirrhosis.

## 1. Introduction

In recent decades, alcohol consumption, which has been on the rise in many parts of the world, is a key factor that explains the constant upward trend of mortality associated with this etiology. The World Health Organization estimated in 2018 that 48% of the people diagnosed with liver cirrhosis died due to alcohol abuse [1]. The effects of alcohol consumption on the general population have been confirmed by recent studies. If specific precautions are not put in place, alcoholic liver disease-related mortality will increase by up to 75% by 2040, according to a study by Julien et al. Additionally, it is estimated that more than a million individuals will die from this ailment in the next 20 years, with 35% of those deaths occurring in subjects under the age of 55. Moreover, it is anticipated that the rate of decompensation of alcoholic liver cirrhosis and development of hepatocarcinoma will increase by more than 65%. Of note is the fact that if the percentage of high-risk drinking could be decreased by as little as 3%, nearly one-third of alcohol-related deaths could be avoided [2].

Currently, this pathology has become a problem with a major impact on public health in the USA, representing the twelfth cause of death [3]. Alcohol-related cirrhosis, the leading indication for liver transplantation in this country, is responsible for a considerable number of deaths among the young population (25–34 years). Despite its influence on mortality, ALC is associated with significant economic consequences. According to budgetary reports in the United States, five billion dollars was allocated for the care of patients with alcoholic liver cirrhosis [4,5,6,7].

In Europe, the prevalence of the disease is significantly higher, the majority being reported in countries from the western and central areas, where the mortality level has reached up to 9.2/100,000 inhabitants [8,9]. Although chronic alcohol consumption has decreased progressively in recent years, approximately one-third of individuals older than 15 years declare at least one episode of excessive alcohol use every month, in which Romania ranks in second place, with a rate of 35%, far above the share recorded in the EU (20%) [10,11]. With an upward trend in the prevalence (from 96.3 cases/100,000 inhabitants in 1990 to 118.3 cases/100,000 inhabitants in 2017), alcoholic liver cirrhosis represents a serious threat, and Romania is an example that illustrates the trend of the phenomenon in central Europe [12]. The worsening of liver dysfunction, which leads to the development of multiple complications, is responsible for numerous hospitalizations and has a strong socio-economic impact on patients, families, and healthcare systems [13]. The implementation of the Baveno guideline and surveillance strategies has resulted in a significant decrease of decompensation of liver disease due to variceal bleeding. Worldwide, numerous studies reported that ascites, infections, hepatic encephalopathy, and hepatorenal syndrome are the current challenges that have an important effect on the morbidity of ALC [14]. Better risk stratification is essential to guide the clinical approach and select the most appropriate therapeutic measures to increase life expectancy.

In this context, we aimed to evaluate the predictive role of complications on mortality in ALC, with the examination of their relationship with survival prognosis.

## 2. Materials and Methods

**Figure 1:**
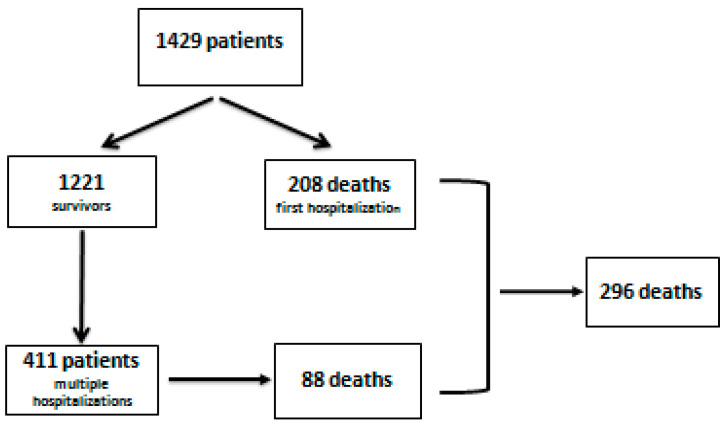
Flow chart of patient selection.

We retrospectively studied 1429 patients who were hospitalized between January 2019 and April 2022 at the Institute of Gastroenterology and Hepatology Iasi, Romania. There were 296 deaths recorded, most of them being reported during the first hospitalization (208). Among the 411 patients who had multiple hospitalizations, 88 people died (Figure 1). The inclusion criteria consist of a diagnosis of alcoholic liver cirrhosis for the first time, based on clinical, imagistic, and laboratory data, and hospitalization for an episode of vascular or parenchymal decompensation.

The patients who did not have relevant information in the medical files (laboratory data, endoscopic or imaging studies) and had a hospitalization of less than 24 h were excluded from the study. Figure 1 presents the flowchart of patient selection in our research study:

Demographic, clinical, paraclinical, and imaging data were collected from the electronic medical records of the patients. We recorded the complications developed during hospitalization, the association of viral hepatitis (hepatitis virus B or hepatitis virus C), condition at discharge, and cause of death. The scores from the model for end-stage liver disease-sodium (MELD-Na) and Child–Turcotte–Pugh (CTP) were calculated during the first hospitalization to assess the severity of the disease. The CTP score was determined according to the presence of ascites, hepatic encephalopathy, bilirubin concentrations, serum albumin, and the level of prothrombin time. Using an online scoring calculator (MDcalc MELD-Na), the values for MELD were initially determined based on the formula: 10 × (0.957 × ln [creatinine, mg/dL]) + (0.378 × ln [bilirubin, mg/dL] + (1.12 × ln [INR]) + 6.43. Subsequently, the MELD-Na score was then calculated using the following equation: MELD-Na seric − [0.025 × MELD × (140 − Na seric)] + 140 [10]. The main complications of ALC identified in this study included hepatic encephalopathy, ascites, jaundice, variceal bleeding, spontaneous bacterial peritonitis, and hepatorenal syndrome. Infections, sepsis, acute alcoholic hepatitis, and hepatocarcinoma also had a significant impact on survival. Ascites was clinically identified based on the increased volume of the abdomen and imaging methods (ultrasound or computer tomography) being confirmed by paracentesis. It was considered as a decompensation of the liver disease, grade 2 and 3 of ascites. Jaundice was diagnosed according to the color changes of the skin and sclera as the consequence of the increased blood level of total bilirubin above 2–2.5 mg/dL [15]. For the classification of hepatic encephalopathy, the West Haven criteria were used, with the diagnosis being established according to the clinical examination and the laboratory values of ammonia. Esophageal and/or gastric variceal bleeding was determined based on clinical findings (hematemesis, melena) determined by an upper gastrointestinal endoscopy [16]. According to the EASL guidelines, spontaneous bacterial peritonitis (SBP) was defined as a neutrophil count ≥ 250 cells/mm^3^, with or without a positive bacterial culture of ascitic fluid collected from a patient without an abdominal source of infection [16]. The criteria used to establish sepsis included the detection of a newly diagnosed dysfunction of an organ, combined with an increase of a sequential organ failure assessment (SOFA score) of more than 2 points associated with a suspected or proven infection. Infections were identified based on the positive result of culture collected from blood, urine, feces, or sputum samples, chest imaging, urine microscopy, or clinical examination compatible with the development of infection [15]. The establishment of acute alcoholic hepatitis relied on the recent onset of jaundice, with or without other signs of liver decompensation (ascites and/or encephalopathy) in individuals who declared heavy alcohol consumption. To diagnose and stage hepatocarcinoma in patients with liver cirrhosis of alcoholic etiology, the level of alpha-fetoprotein, liver function, and imaging evaluation by abdominal ultrasound and computer tomography, as well as the clinical performance status was useful [17]. Hepatorenal syndrome was characterized by the progressive increase in the serum creatinine concentration in patients with chronic liver disease who did not respond to the discontinuation of diuretics and volume expansion with albumin [15].

Statistical analyses were performed using IBM SPSS, Version 28.0 (IBM SPSS Inc., Chicago, IL, USA). Quantitative variables were expressed as mean and standard deviation (SD), and qualitative variables as number and frequency. Chi-square and *t*-Student tests were used to compare qualitative and quantitative variables between deceased patients and survivors. Using the ROC graphical representation, the performance of the CTP and MELD-Na prognostic scores was evaluated according to the calculated values for the area under the curve (AUROC), sensitivity, specificity, and positive and negative predictive values for the cut-off. Univariate analysis was used to determine the possibility of a complication to represent a risk factor for death. Only the complications that had statistical significance (*p* < 0.05) were included in the multivariate analysis to confirm the independent predictors of mortality. The survival probability of patients was estimated by Kaplan–Meier analysis and the differences were examined comparatively using the log-rank test.

## 3. Results

The average age of the 1429 patients who met the criteria for inclusion in this study was 56.15 ± 11.49 years, with variations between 25–92 years and similar distributions in the group of survivors (*n* = 1133; 56.05 ± 11.61 years/25–92 years) and the deceased (*n* = 296; 55.18 ± 10.77 years/27–83 years) (*p* = 0.079), respectively. In our cohort, there were more men than women with a ratio of 2:1 (survivors: 68%; deceased: 66.6%; *p* = 0.625), with no significant statistical difference when analyzing their residence (urban versus rural; *p* = 0.506). On the other hand, most of the patients were diagnosed only with ALC (survivors: *n* = 903; 79.7%; deceased: *n* = 256; 86.5%) when compared to those who had associated viral hepatitis B (survivors *n* = 112; 9.9%; deceased: *n* = 17; 5.7%) or C infection (survivors: *n* = 118; 10.4%; deceased: *n* = 23; 7.8%). Concerning the clinical signs of the decompensation of ALC between the two groups (survivors versus deceased) hepatic encephalopathy (70.6%; *p* < 0.001), ascites (48.6%; *p* = 0.004), and variceal bleeding (32.1%; *p* < 0.001) were more frequently reported in the latter cohort.

Moreover, there was also a tendency towards higher rates of infectious complications (37.8%; *p* < 0.001), mainly spontaneous bacterial peritonitis (18.6%; *p* < 0.001) and sepsis (15.9%; *p* < 0.001), but also for the occurrence of hepatorenal syndrome (11.5%; *p* < 0.001) or hepatocellular carcinoma (7.1%; *p* = 0.001). In the case of the other variables, no significant differences were observed (Table 1). In order to evaluate the severity of liver dysfunction, it was found that the CTP class C score was established more frequently in deceased patients (*n* = 258; 87.2%), the number being twice as high than in those who survived (*n* = 477; 42.1%) (*p* < 0.001). The average values calculated for the two prognostic scores used were significantly higher in the deceased group (CTP: 12.1 ± 1.33 versus 8.92 ± 2.37, respectively, MELD-Na: 23.75 ± 6.73 versus 15.52 ± 7.08) (*p* < 0.001). Table 1 presents the clinical and demographic data of the investigated group.

### 3.1. First Admission

A mortality rate of 14.6% (*n* = 208 deaths out of 1429 patients) was recorded after the first admission. The complications of the disease, univariately analyzed, negatively affected the survival rate, with significant values being linked to infections (sepsis: OR = 21.98; *p* < 0.001; spontaneous bacterial peritonitis: OR = 11.94; *p* < 0.001) and hepatorenal syndrome (OR = 9.35; *p* < 0.001), which increased the risk of death at least 10–20 times. Moreover, a strong correlation was also observed between hepatic encephalopathy (OR = 3.47; *p* < 0.001), hepatocellular carcinoma (OR = 3.92; *p* < 0.001), variceal bleeding (OR = 2.47; *p* < 0.001), other types of infections (OR = 2.43; *p* < 0.001), or ascites (OR = 1.49; *p* = 0.009) and a negative clinical outcome (Table 2).

The independent predictors confirmed by multivariate analysis were the association of variceal bleeding and infections with hepatic encephalopathy (R^2^ adjusted = 0.227; *p* < 0.001) or ascites (R^2^ adjusted = 0.196; *p* < 0.001), each combination being responsible for two out of 10 of deaths during the first admission. After adding the diagnosis of hepatocellular carcinoma and hepatorenal syndrome (R^2^ adjusted = 0.276; *p* < 0.001), the number of deaths registered a small increase (<8%).

We also evaluated the main reason of admission to the hospital in our cohort, at first and second presentation. We found that, regarding the first admission, the main reason for hospitalization was the presence of ascites (63.2%). Concerning the second admission, the most frequent cause of hospitalization was variceal bleeding (54.9%).

Regarding first admission, we performed a *t*-test for equality of means in order to observe if there were significant statistical differences between complications of survivors and the deceased. The results proved that there were no differences for SBP (*t*= −4.958, *p* = 0.001), sepsis (*t* = −4.436, *p* = 0.001), and HRS (*t* = −4.097, *p* = 0.001). However, the other complications were relevant during first hospitalization: ascites (*t* = 0.172, *p* = 0.864), jaundice (*t* = 1.848, *p* = 0.078), variceal bleeding (*t* = 1.145, *p* = 0.157), other infections (*t* = −0.934, *p* = 0.350), EH (*t* = −0.592, *p* = 0.054), acute alcoholic hepatitis (*t* = 0.823, *p* = 0.411), and hepatocellular carcinoma (*t* = −0.856, *p* = 0.392).

**Figure 2:**
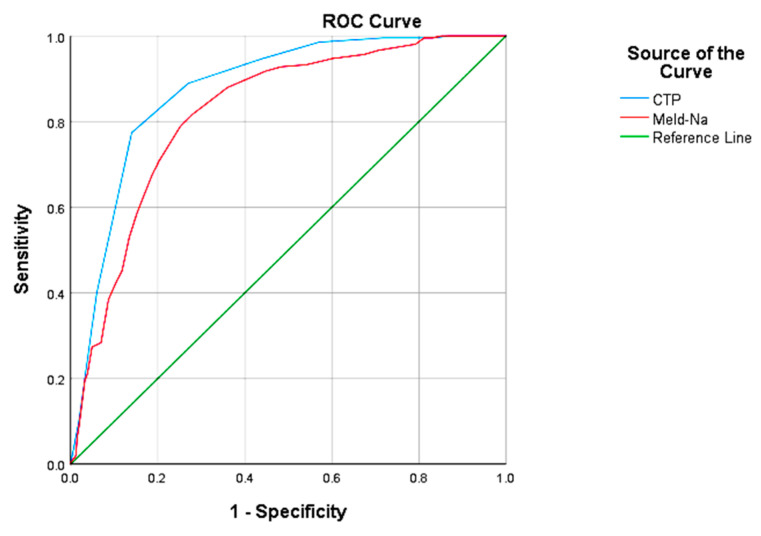
Analysis of the ROC curve for predicting scores (CTP and MELD-Na). CTP: Child–Turcotte–Pugh, MELD-Na: model for end-stage liver disease-sodium.

The performance of the scores used to assess liver dysfunction (CTP and MELD-Na) was analyzed based on the ROC curve. The results measured for the area under the curve were extremely close (CTP: AUROC 0.876; CI 95% 0.854–0.898; *p* < 0.001; MELD-Na: AUROC 0.822; CI 95% 0.794–0.850; *p* < 0.001) (Figure 2).

Although the CTP was sensitive (89%), the specificity was much lower (64%) for a cutoff of 10.5. Instead, the MELD-Na score had a good balance between the two parameters (cut-off = 19; sensitivity 89% and specificity 83%), with a predictive positive value (PPV) approximately twice as high when compared to the CTP (58% versus 31%), which increases its accuracy (Table 3).

### 3.2. Multiple Admissions

From the investigated group (*n* = 1429), only 411 patients had multiple hospitalizations, 88 of which deceased (6.2%). The main factors associated with mortality in univariate analysis were infections (OR = 18.71; *p* < 0.001; spontaneous bacterial peritonitis: OR = 11.33; *p* < 0.001), hepatorenal syndrome (OR = 6.79; *p* < 0.001), recurrent episodes of hepatic encephalopathy (OR = 2.84; *p* < 0.001), and variceal bleeding (OR = 2.60; *p* = 0.001). In the case of the other variables, no significant differences were observed, as can be seen in Table 4.

The model including variceal bleeding and infections with hepatic encephalopathy (R^2^ adjusted = 0.02; *p* = 0.004), as indicated by multivariate analysis for multiple hospitalizations, had a 10 times lower impact on mortality compared to that identified for patients during the first admission (R^2^ adjusted = 0.227; *p* < 0.001).

We further evaluated the Child–Pugh score for all patients at the second admission in the hospital and have seen a slight increase from the first presentation, namely 9.84 ± 2.75 (*p* = 0.038). The same trend can be observed regarding the MELD-Na score, with an increase up to 19.1 ± 6.94 from 15.52 ± 7.08 (*p* = 0.025). These changes in the scores are most likely due to the fact that most of the patients with alcoholic liver cirrhosis continue to drink alcohol even after the first decompensation of the liver disease.

**Figure 3:**
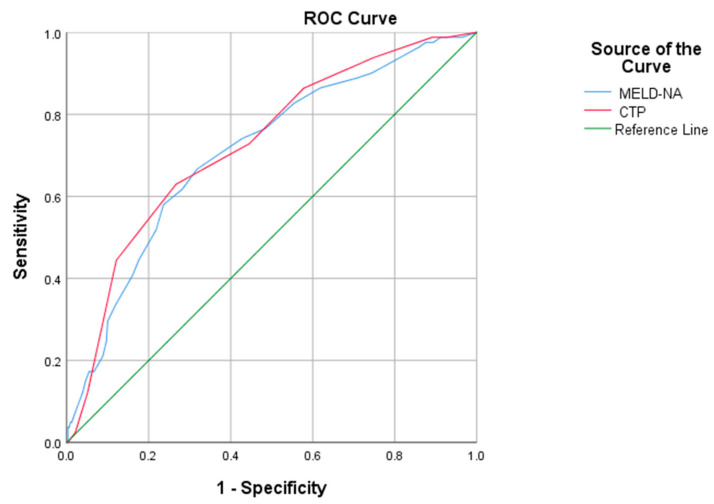
Analysis of the ROC curve for predicting scores (CTP and MELD-Na). CTP: Child–Turcotte–Pugh, MELD-Na: model for end-stage liver disease-sodium.

Regarding patients with multiple admissions, CTP and Meld-Na scores showed a slightly reduced value of AUROC, compared with the values for the patients in the first admission (Figure 3, Table 5).

Based on the interaction between the complications and the survival status, we determined through linear regression that, within patients with a single complication of the disease, we could identify two predictors of survival: variceal bleeding (β=−0.337, *p* = 0.001) and infections (β=−0.214, *p* = 0.017). Among patient with multiple complications, relevant factors for survival also included hepatic encephalopathy (β=−0.261, *p* = 0.002) and SBP (β=−0.186, *p* = 0.027).

The Kaplan–Meier analysis and the log-rank test proved that there were significant differences between the survival of patients with multiple admissions. It was found that sepsis (mean survival period 13.4 months; CI 95%: 8.7–18.2; *p* < 0.001) and spontaneous bacterial peritonitis (mean survival period 16.9 months; CI 95%: 12.9–20.9; *p* < 0.001) negatively influenced the prognosis, having the lowest average restricted periods.

The survival rate at 6 months for the two predictors had similar values (62.8%, respectively, 66.7%), with a significantly important decline for sepsis compared to spontaneous bacterial peritonitis recorded at 12 months (32.1% and 44.9%, respectively) and at 18 months (20.6% and 34.2%, respectively). Regarding the hepatorenal syndrome, the mean survival period was 20.2 months (CI 95%: 15.3–25.0; *p* < 0.001). Moreover, after 12 months, the survival rate was approximately two times lower (46.4%) compared with those who were not diagnosed with this condition.

**Figure 4:**
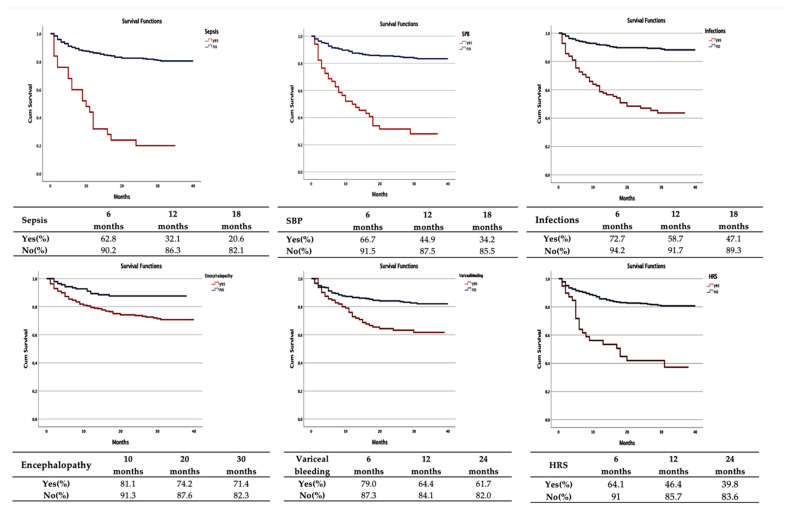
Evolution of alcoholic liver cirrhosis in patients with multiple admissions—Kaplan–Meier curve. SBP: spontaneous bacterial peritonitis HRS: hepatorenal syndrome.

On the opposite side, the longest interval until the occurrence of death belongs to hepatic encephalopathy (31.2 months; CI 95%: 29.4–32.9; *p* < 0.001), but the survival rates at 10, 20, and 30 months (81.2%, 74.2%, and 71.4%, respectively) had similar results to those recorded in patients who were not diagnosed with this complication (91.3%, 87.6%, and 82.3%, respectively) (Table 6 and Figure 4).

## 4. Discussion

Establishing the prognosis is an essential step for the initial evaluation of any disease because it allows a better risk stratification, while playing a role in guiding the clinical approach and selecting the most appropriate specific therapeutic measures for each case. In this retrospective study, 1429 patients who were diagnosed with alcoholic liver cirrhosis were included, registering a mortality rate of 20.7%, close to the value reported by Bhattarai et al. (19.8%) [18]. A total of 411 patients presented multiple admissions (28.8% of the investigated group). This result is much lower than the readmission rates reported by various researchers (50–78%), possibly due to the limitation of the patients’ accessibility to medical services during the COVID-19 pandemic [19,20,21]. Hepatic encephalopathy (70.6%), ascites (48.6%), and variceal bleeding (32.1%) were the most common causes that led to hospitalization. Similar findings were reported by Bhattacharyya et al., who presented a clinical profile associated with the hospitalization of this category of patients, dominated by variceal bleeding (47.8%) and hepatic encephalopathy (39.1%) [22]. Instead, ascites was the main indicator of severe prognosis among patients with alcoholic cirrhosis in the study by Bhattarai et al., followed by variceal bleeding (42.3%) and hepatic encephalopathy (32.5%) [23].

Based on the univariate analysis, nine risk factors were identified to be significantly associated with an unfavorable prognosis of the patients during first admission. Among them, infections, both spontaneous bacterial peritonitis and sepsis, as well as hepatorenal syndrome stand out as the main predictors that increased the risk of death at least 10–20 times. Also, a strong correlation was highlighted for hepatic encephalopathy, hepatocarcinoma, variceal bleeding, and, to a lesser extent, ascites (Table 2). A similar situation was presented by Reddy et al., who estimated an increased risk of mortality for hepatic encephalopathy (OR = 2.24; *p* < 0.01), spontaneous bacterial peritonitis (OR = 2.28 95%; *p* < 0.01), and ascites (OR = 1.76; *p* < 0.01), but with lower values compared to our study, probably due to the fact that the group studied only had 325 patients [18].

The importance of the risk factors identified in the univariate analysis was confirmed by the multivariate analysis. Six independent predictors were included that had a significant statistical value in the univariate analysis. It has been shown that there was a strong correlation between the presence of variceal bleeding and infections. By adding hepatic encephalopathy or ascites, each model was responsible for approximately two out of 10 deaths during the first admission. These results allow for a better risk stratification, while playing a role for guiding the clinical approach and selecting the most appropriate therapeutic measures necessary to reduce in-hospital mortality and increase life expectancy.

It was estimated that decompensated liver cirrhosis was associated with a 9.7 times higher risk of death compared to the general population and, for this reason, prognostic score systems have been used and are extremely useful for disease stratification [23]. In this study, we used two scores (CTP and MELD-Na) to assess liver dysfunction. We took into account the results of a systematic review performed by Cholongitas et al., which showed that the CTP score remains particularly useful for the evaluation of liver disease in daily clinical practice, while the MELD-Na is considered an important prognostic tool for the terminal stage of the disease [24,25].

Our analysis supports the validity of these two scores as useful predictors of mortality in patients with decompensated alcoholic liver cirrhosis in line with data provided by Papatheodoridis et al. [26]. Of note, we found that the CTP class C score was twice as frequent in deceased patients compared to those who survived (87.2% versus 42.1%) (*p* < 0.001). Also, the mean values calculated for these scores were significantly higher for the group of deceased patients (CTP: 12.1 ± 1.33 versus 8.92 ± 2.37, respectively, MELD-Na: 23.75 ± 6.73 versus 15.52 ± 7.08, respectively) (*p* < 0.001).

The same was demonstrated by Jain et al., who stated that patients with liver cirrhosis of alcoholic etiology have less chance to survive for one year, if at the time of hospitalization the values for the MELD-Na and CTP are higher than 20.9 ± 6.9 and 10.3 ± 1.86, respectively [15,27].

To validate the results of this study, we compared the performance of the two scores according to the ROC curve analysis. The increased values we obtained for AUROC (CTP: 0.876; MELD-Na: 0.822) and sensitivity (CTP: 89%; MELD-Na: 89%) were strongly correlated with the role of the predictors of hospital mortality. The impact of these scores was studied by Dupont et al. on a group of 281 patients with cirrhosis admitted to the intensive care unit, who reported a mortality rate of 25.3% in a direct causal relationship with the important dimensions that were measured for the area under the curve (MELD-Na: AUROC 0.81; 95% CI: 0.76–0.87, respectively, CTP: AUROC 0.76; 95% CI: 0.70–0.82) [28]. On the other hand, we noticed that there was an increased level of accuracy regarding the MELD-Na score, determined by a much higher specificity (83% versus 64%) and an almost double positive predictive value (PPV) (58% versus 31%), results comparable to those presented in previous studies (Table 7) [29,30,31].

We obtained a cut-off value of 17 for the MELD-Na score, relatively lower than that reported in other studies [32,33]. While most specialists focused on patients with end-stage alcoholic cirrhosis or hospitalized in an intensive care unit and scheduled for liver transplantation, we evaluated all cirrhotic subjects, regardless of the severity of the disease [33,34,35].

Readmissions represented a valuable opportunity to improve the care of patients with alcoholic liver cirrhosis. Therefore, it was extremely important to understand the reasons for readmission in order to establish a strategy that can be implemented to reduce this rate. Consistent with other studies, we identified hepatic encephalopathy, infections, and variceal bleeding as the main causes of multiple admissions [36,37,38]. Unfortunately, the model of the association of these three independent predictors, as revealed by the multivariate analysis for multiple hospitalizations (R^2^ adjusted = 0.02; *p* = 0.004), could not be validated because it had a 10 times smaller impact on mortality compared to that corresponding to patients at the first diagnosis of liver disease (R^2^ adjusted = 0.227; *p* < 0.001).

It was unanimously accepted that patients with liver cirrhosis present a major risk for the development of infections due to the state of immunosuppression and increased intestinal permeability that favors bacterial translocation [39]. Currently, these complications have generated great interest because they are considered triggers of acute liver failure syndrome, the most common cause of death in patients with decompensated alcoholic liver cirrhosis [40,41]. Regardless of the severity of the disease, infections were associated with a severe prognosis that decrease short-term survival [42]. Due to the multifactorial mechanisms of the exponential increase in the incidence of multiresistant microorganisms in the hospital environment, Grgurevica et al. showed that the cases of infectious complications in the decompensation of alcoholic liver cirrhosis were almost double compared to non-alcoholic patients (76.2% versus 45.8%), the most common being spontaneous bacterial peritonitis and sepsis, which was similar with the results of our study [43].

Based on the Kaplan–Meier analysis, we concluded that sepsis and spontaneous bacterial peritonitis had the lowest values for the mean restrictive period (13.4 months; CI 95%: 8.7–18.2 versus 16.9 months; CI 95%: 12.9–20.9). Although the survival rate at 6 months for the two predictors had significantly close values (62.8% and 66.7%, respectively), at 12 months and 18 months, a downward slope was recorded, especially for sepsis (32.1% and 20.6%, respectively) versus spontaneous bacterial peritonitis (44.9% and 34.2%, respectively).

This study has several important limitations beyond its retrospective nature. It was carried out in a single medical institution, which could underestimate the real value of the specific morbidity. The lack of validation of models of mortality in the case of multiple readmissions and the lack of a possibility of following the evolution of some patients after discharge from our hospital represent two other disadvantages that we found in this analysis. This approach made an update of the data related to the impact of liver cirrhosis in Romania during the investigated period, which proved several advantages. Firstly, it is based on real-life information with all patients were studied in the same way by the same team of gastroenterologists and does not represent data from national statistical databases that are dependent on correct coding. Secondly, two models were established that can predict first hospital admission mortality, which could help in risk stratification, defining priorities and strategies for improving population health care.

## 5. Conclusions

The prognosis of the disease is negatively influenced by the worsening of liver dysfunction and the occurrence of complications. In this study, we found that the main predictors for mortality are infections, hepatic encephalopathy, variceal bleeding, and hepatorenal syndrome. Improving compliance and strict application of specific follow-up and treatment strategies could contribute to a better prognosis of patients with alcoholic liver cirrhosis, with reduced readmission rates and mortality.

## Figures and Tables

**Table 1:** Demographic and clinical data of the investigated group.

Variables	Patients	Survivors	Deceased	*p*
**Patients** *n* (%)	1429 (100%)	1133 (79.3%)	296 (20.7%)	-
**Age** (average ± SD)	56.15 ± 11.49	56.05 ± 11.61	55.18 ± 10.77	0.079
(limits/median)	(25–92/57)	(25–92/57)	(27–83/58)	
**Gender**				0.625
Male, *n* (%)	968 (67.7%)	771 (68.0%)	197 (66.6%)	
Female, *n* (%)	461 (32.3%)	362 (32.0%)	99 (33.4%)	
**Residence**				0.506
Urban, *n* (%)	623 (43.6%)	499 (44.0%)	124 (41.9%)	
Rural, *n* (%)	806 (56.4%)	634 (55.0%)	172 (58.1%)	
**Etiology**				
Alcohol, *n* (%)	1159 (81.1%)	903 (79.7%)	256 (86.5%)	
Alcohol + B virus, *n* (%)	129 (9.0%)	112 (9.9%)	17 (5.7%)	**0.024**
Alcohol + C virus, *n* (%)	141 (9.9%)	118 (10.4%)	23 (7.8%)	
**Complication**				
**Ascites,***n* (%)	589 (41.2%)	445 (39.3%)	144 (48.6%)	**0.004**
**Jaundice,***n* (%)	191 (13.4%)	142 (12.5%)	49 (16.6%)	0.070
**Digestive bleeding**				
Variceal, *n* (%)	292 (20.5%)	197 (17.4%)	95 (32.1%)	**<0.001**
Non-variceal, *n* (%)	109 (7.6%)	88 (7.8%)	21 (7.1%)	0.698
**Infections,***n* (%)	209 (14.6%)	97 (8.6%)	112 (37.8%)	**<0.001**
SBP, *n* (%)	87 (6.1%)	32 (2.8%)	55 (18.6%)	**<0.001**
Sepsis, *n* (%)	63 (4.4%)	16 (1.4%)	47 (15.9%)	**<0.001**
**Other infections,***n* (%)	90 (6.3%)	56 (4.9%)	34 (11.5%)	**<0.001**
**Encephalopathy,***n* (%)	713 (49.9%)	504 (44.5%)	209 (70.6%)	**<0.001**
**Hepatic carcinoma,***n* (%)	55 (3.8%)	34 (3.0%)	21 (7.1%)	**0.001**
**Hepatorenal syndrome,***n* (%)	59 (4.1%)	25 (2.2%)	34 (11.5%)	**<0.001**
**Acute alcoholic hepatitis,***n* (%)	112 (7.8%)	89 (7.9%)	23 (7.8%)	0.961
**Prognostic score**				
**CTP Class**				
**A***n* (%)	207 (14.5%)	205 (18.1%)	2 (0.7%)	**<0.001**
**B***n* (%)	487 (34.1%)	451 (39.8%)	36 (12.2%)	**<0.001**
**C***n* (%)	735 (51.4%)	477 (42.1%)	258 (87.2%)	**<0.001**
**CTP** (average ± SD)	9.37 ± 2.50	8.91 ± 2.37	12.10 ± 1.33	**<0.001**
(limits/median)	(5–15/10)	(5–15/9)	(6–15/12)	
**MELD-Na** (average ± SD)	17.41 ± 7.72	15.52 ± 7.08	24.94 ± 6.28	**<0.001**
(limits/median)	(5–43/17)	(5–40/15)	(8–43/25)	

SBP: spontaneous bacterial peritonitis, CTP: Child–Turcotte–Pugh, MELD-Na: model for end-stage liver disease-sodium.

**Table 2:** Predictive factors—first hospitalization/univariate analysis.

Variables	Deceased (*n* = 208)	Survivors (*n* = 1221)	Odds Ratio (CI 95%)	*p*
**Variceal bleeding***n* (%)	73 (35%)	219 (18.0%)	2.47 (1.80–3.41)	**<0.001**
**Non-variceal bleeding***n* (%)	14 (6.8%)	95 (7.8%)	0.86 (0.48–1.53)	0.527
**Infections***n* (%)	100 (49.0%)	109 (8.9%)	9.45 (6.75–13.21)	**<0.001**
**SBP***n* (%)	53 (25.5%)	34 (2.8%)	11.94 (7.52–18.95)	**<0.001**
**Sepsis***n* (%)	47 (22.6%)	16 (1.3%)	21.98 (12.18–39.69)	**<0.001**
**Other infections***n* (%)	25 (12.0%)	65 (5.3%)	2.43 (1.41–2.91)	**<0.001**
**Hepatic encephalopathy***n* (%)	155 (74.5%)	558 (45.7%)	3.47 (2.49–4.84)	**<0.001**
**Ascites***n* (%)	103 (49.5%)	486 (39.8%)	1.48 (1.10–1.99)	**0.009**
**Jaundice***n* (%)	33 (15.9%)	159 (13.0%)	1.26 (0.84–1.89)	0.267
**Hepatocellular carcinoma***n* (%)	21 (10.1%)	34 (2.8%)	3.92 (2.23–6.90)	**<0.001**
**Acute alcoholic hepatitis***n* (%)	13 (6.3%)	99 (8.1%)	0.76 (0.42–1.37)	0.919
**Hepatorenal syndrome***n* (%)	34 (16.3%)	25 (2.0%)	9.35 (5.45–16.07)	**<0.001**

SBP: spontaneous bacterial peritonitis.

**Table 3:** Performance of predicting scores (CTP and MELD-Na)—first hospitalization.

Scor	AUROC	*p*	CI 95%	Cutoff	Sensitivity	Specificity	PPV	NPV
**CTP**	0.876	0.001	0.854–0.898	10.5	89%	64%	31%	63%
**MELD-Na**	0.822	0.001	0.794–0.850	19	88%	83%	58%	64%

PPV: positive predictive value; NPV: negative predictive value; AUROC: area under the ROC curve.

**Table 4:** Risk of death depending on complications—multiple hospitalizations.

Variabile	Deceased (*n* = 88)	Survivors (*n* = 323)	Odds Ratio	CI 95%	*p*
**Non-variceal bleeding***n* (%)	6 (6.8%)	21 (6.5%)	1.05	0.41–2.69	0.915
**Variceal bleeding***n* (%)	39 (44.3%)	74 (22.9%)	2.68	1.63–4.39	**0.001**
**Infections***n* (%)	56 (63.6%)	55 (17.0%)	8.53	5.06–14.38	**<0.001**
**SBP***n* (%)	34 (38.6%)	17 (5.3%)	11.33	5.92–21.71	**<0.001**
**Sepsis***n* (%)	20 (22.7%)	5 (1.5%)	18.71	6.78–51.58	**<0.001**
**Other infections***n* (%)	13 (14.8%)	38 (11.8%)	1.30	0.70–2.56	0.449
**Hepatic encephalopathy***n* (%)	72 (81.8%)	198 (61.3%)	2.84	1.58–5.11	**<0.001**
**Ascites***n* (%)	54 (61.4%)	182 (56.3%)	1.23	0.78–1.99	0.843
**Jaundice***n* (%)	17 (19.3%)	38 (11.8%)	1.79	0.96–3.37	0.068
**Hepatocellular carcinoma***n* (%)	6 (6.8%)	30 (9.3%)	0.71	0.29–1.78	0.450
**Hepatorenal syndrome***n* (%)	23 (26.1%)	16 (5.0%)	6.79	3.40–13.56	**<0.001**
**Acute alcoholic hepatitis***n* (%)	3 (3.4%)	14 (4.3%)	0.78	0.22–2.77	0.385

SBP: spontaneous bacterial peritonitis.

**Table 5:** Performance of predicting scores (CTP and MELD-Na)—multiple hospitalizations.

Score	AUROC	*p*	CI 95%	Cutoff	Sensitivity	Specificity	PPV	NPV
**CTP**	0.726	0.001	0.666–0.787	10.5	86%	57.9%	19.7%	80.29%
**MELD-Na**	0.709	0.001	0.647–0.772	19	82.7%	55.5%	19.7%	80.29%

**Table 6:** The average survival period in patients with alcoholic liver cirrhosis depending on complications.

Variables	Deaths	Average Survival Period/ CI 95% (Months)	Log-Rank Test
**Patients**	88	-	-
**Variceal bleeding**	39	27.8 (24.9–30.7)	**<0.001**
**Infections**	56	21.4 (18.5–24.3)	**<0.001**
**SBP**	34	16.9 (12.9–20.9)	**<0.001**
**Sepsis**	20	13.4 (8.7–18.2)	**<0.001**
**Hepatic encephalopathy**	72	31.2 (29.4–32.9)	**<0.001**
**Ascites**	54	32.3 (30.4–34.1)	0.464
**Other infections**	13	28.2 (24.3–32.2)	0.296
**Jaundice**	17	32.3 (27.9–36.6)	0.055
**Non-variceal bleeding**	6	28.2 (24.9–32.7)	0.990
**Hepatorenal syndrome**	23	20.2 (15.3–25.0)	**<0.001**
**Hepatocelular carcinoma**	6	32.6 (29.3–35.9)	0.366
**Acute alcoholic hepatitis**	3	305 (25.6–35.4)	0.670

**Table 7:** Score values in patients with alcoholic liver cirrhosis.

Authors	Score	AUROC	Cutoff	Sensitivity	Specificity	PPV	NVP
Radisavljevic et al., 2017 [31]	CTP	0.861	11.50	85%	66.7%	-	-
	MELD-Na	0.814	22.1	83.3%	70%	-	-
Abbasy et al., 2022 [29]	CTP	0.709	9	90.7%	29.13%	54.7%	76.9%
	MELD-Na	0.717	21.93	94.5%	40.7%	57.5%	87.5%
Fayad et al., 2015 [30]	MELD-Na	0.781	20	70%	79%	49%	90%

PPV: positive predictive value; NPV: negative predictive value; AUROC: area under the ROC curve.

## Data Availability

The data presented in this study are available on request from the corresponding author. The data are not publicly available because they are the property of the Institute of Gastroenterology and Hepatology, Iasi, Romania.
